# Impact of the Exercise Right for Active Ageing program on physical function in older adults: a quasi-experimental pre-post study

**DOI:** 10.1186/s12877-023-04499-5

**Published:** 2023-12-04

**Authors:** Christina Ekegren, Helen Skouteris, Darshini Ayton, Sze-Ee Soh

**Affiliations:** 1 Rehabilitation, Ageing and Independent Living (RAIL) Research Centre, School of Primary and Allied Health Care, Monash University, 47-49 Moorooduc Hwy, Frankston, VIC 3199 Australia; 2 Health and Social Care Unit, School of Public Health and Preventive Medicine, Monash University, Monash, Australia; 3 Department of Physiotherapy, School of Primary and Allied Health Care, Monash University, Monash, Australia

**Keywords:** Older adults, Physical activity, Mobility, Balance, Strength

## Abstract

**Background:**

The Exercise Right for Active Ageing (ERAA) program was established to improve access to exercise classes for community-dwelling older Australians. The aims of this study were to determine whether older adults, who participated in ERAA exercise classes experienced a change in physical function, and identify factors associated with this change.

**Methods:**

Participants included community-dwelling older adults, aged ≥ 65 years, from every state and territory of Australia. The ERAA program included 12 subsidised, weekly, low- to moderate-intensity exercise classes, delivered by accredited exercise scientists or physiologists (AESs/AEPs). Primary outcomes included the 30 s Sit-to-Stand (STS) and the 3-metre Timed Up and Go (TUG) tests. Secondary outcomes included grip strength, the Chair Sit and Reach test, and waist circumference. Linear mixed-effects regression models were used to evaluate the change in outcomes following program completion, and to determine factors associated with changes in the primary outcomes.

**Results:**

3,582 older adults (77% female) with a median (IQR) age of 72 (69–77) years completed follow-up testing. For all primary and secondary outcomes, there was a statistically significant improvement after program completion (p < 0.001). The STS increased by 2.2 repetitions (95% CI: 2.1, 2.3), the TUG decreased by 0.9 s (95% CI: -1.0, -0.8), right and left grip strength increased by 1.3 kg (95% CI: 1.2, 1.5) and 1.5 kg (95% CI: 1.3, 1.6), respectively, right and left reach increased by 1.7 cm (95% CI: 1.4, 2.0), and waist circumference decreased by 1.2 cm (95% CI: -1.4, -1.1). Greater improvements in STS were observed for participants aged 65–69 years, females, and those with greater socio-economic disadvantage. For the TUG, greater improvements were observed in participants reporting 2 + comorbidities, and residing in outer regional areas and areas with greater socio-economic disadvantage.

**Conclusions:**

Participation of older Australians in the ERAA program, led to statistically significant improvements in physical function. The program reached a large number of older Australians from every state and territory, including those from regional and remote parts of Australia, aged over 85 years, and with high levels of comorbidity, which supports the feasibility and acceptability of AES- and AEP-led exercise classes amongst community-dwelling older Australians.

**Trial registration:**

Australian New Zealand Clinical Trials Registry (ANZCTR) (ACTRN12623000483651). Registered 12 May 2023 - Retrospectively registered, https://www.anzctr.org.au/ACTRN12623000483651.aspx.

**Supplementary Information:**

The online version contains supplementary material available at 10.1186/s12877-023-04499-5.

## Background

For community dwelling older adults, the maintenance of physical function is key to sustaining independent living and quality of life [1–3]. It is well established that exercise focussing on aerobic capacity, balance, strength activities and mobility can improve physical function, and also prevent falls, cognitive decline, morbidity and mortality in older adults [4–7]. Yet, as few as 4% of older Australians and 15% of older Americans (aged 75 + years) meet current guidelines on physical activity and strength, which stipulate at least 150 min per week of moderate-intensity physical activity or 75 min per week of vigorous-intensity physical activity (or an equivalent combination of both), and two or more days per week of muscle-strengthening activities [5, 8]. The recent update to the World Health Organization’s (WHO’s) guidelines for older adults (aged 65 + years) further stipulate a minimum of three sessions per week of multicomponent physical activity, comprising balance, strength, endurance, gait and physical function training [9].

A key barrier to older adults meeting physical activity guidelines is the lack of access to suitable and affordable exercise classes in the community [10]. Accordingly, a key recommendation from the WHO’s 2030 Physical activity Global Action Plan is to improve access to exercise options for older adults [11]. Older adults have reported a preference for tailored exercise classes, that can be modified to adapt to any physical limitations and/or disability [12]. Ensuring that classes are accessible, affordable and suitable for individuals with a wide range of physical and cognitive capacities is vital to long term exercise adherence and maintenance of physical function in older adults [10]. Older adults have also expressed a desire for advice from health professionals to facilitate participation in physical activity programs [10].

In Australia, accredited exercise scientists (AESs) and physiologists (AEPs) are university-trained health professionals who use exercise as their main treatment modality, and are trained to develop, and teach exercise classes for people with chronic and complex medical conditions and injuries [13]. They are often employed within community-based fitness and health centres or private clinics, with AEP-delivered services rebatable via Medicare (Australia’s universal health care system, via a general practitioner-referred Chronic Disease Management Plan), the Department of Veterans Affairs, other compensable bodies and most private health funds [14]. Yet, despite the growing number of referrals to AESs and AEPs for exercise prescription [14], there have been few large-scale studies on the effectiveness of AES and AEP-led exercise classes for improving physical function in older adults. In 2019, Exercise & Sports Science Australia (ESSA), Australia’s peak professional organisation for university-trained exercise and sports science practitioners, established the Exercise Right for Active Ageing program [15, 16]. The program aimed to improve access to and affordability of exercise classes for older adults by delivering subsidised, community-based, AES- and AEP-led group exercise classes across Australia.

The primary aim of this study was to determine whether adults aged 65 years and older, who participated in Exercise Right for Active Ageing exercise classes experienced a change in physical function and to identify factors associated with a change in these outcomes. Secondary aims were to determine whether there were changes in grip strength, flexibility (sit and reach) and waist circumference. The primary hypothesis was that older adults would experience significant improvements in physical function following participation in the Exercise Right for Active Ageing program.

## Methods

### Design

This quasi-experimental pre-post study was reported in accordance with the Transparent Reporting of Evaluations with Nonrandomized Designs (TREND) guidelines [17]. Ethical approval was granted by the Monash University Human Research Ethics Committee (Project ID: 21550) and all participants provided written, informed consent. The study was registered with the Australian New Zealand Clinical Trials Registry (ANZCTR) on 12/05/2023 (ACTRN12623000483651).

### Participants

Accredited Exercise Scientists and AEPs employed within fitness centres, community health centres and private clinics from all states and territories of Australia were invited to be part of the Exercise Right for Active Ageing program. Following a nationwide marketing campaign by ESSA, a total of 215 providers joined the program, and were subsequently involved in recruiting participants. Marketing included the establishment of a website [16], with a search function to find a provider, television advertisements for general practices, local community activation events, Facebook advertising, and paper-based materials distributed to health professionals and community organisations. Potential participants self-referred to the provider after exposure to marketing materials. Participants included community-dwelling older adults (aged 65 + years) from across Australia. Exclusion criteria included the inability to participate in a low to moderate-intensity exercise program, in accordance with the Adult Pre-Exercise Screening System (APSS) V2 (Stage 1) (Additional file 1) [18].

### Intervention

The Exercise Right for Active Ageing program ran from August 2019 to June 2022, and consisted of 12 subsidised exercise classes, delivered at a frequency of one class per week, over a maximum period of 16 weeks [19]. Participants paid exercise providers $8.00 per class, with ESSA paying providers an additional $10.91 per participant per class attended. The specific content and delivery were at the discretion of the provider, provided classes were of low to moderate-intensity exercise and suitable for older adults. Class types included falls prevention, strength, and general fitness classes, amongst others (Table 1). Classes were mostly delivered, in-person, at a community-based exercise facility or clinic, although during COVID-19 lockdowns, online delivery options were also made available.

### Measurements

A range of physical performance outcomes were measured before and after program participation (Additional file 1). Primary outcomes included the 30 s Sit-to-Stand (STS) [20], and the 3-metre Timed Up and Go (TUG) tests [21]. Secondary outcomes included grip strength (left and right) [22], the Chair Sit and Reach test (left and right), as an indicator of lower body flexibility [22], and waist circumference, as an indicator of abdominal adiposity [23].

The STS is a measure of lower limb strength and the ability to perform activities of daily living (ADLs) [20]. It is performed by recording the maximum number of times a participant can stand up and sit down from an armless chair in 30 s (secs), with their arms folded across their chest. The TUG is a measure of functional mobility and dynamic balance [21]. It is performed by recording the amount of time taken (in secs) for the participant to stand up from a chair, walk around a cone placed three metres away and return to sitting on the chair. Left and right grip strength was measured in kg using a hand grip dynamometer, with the elbow maintained at waist level [22]. The chair sit and reach test was performed by seating the participant in a chair with limb straight out in front with the ankle dorsi-flexed, asking them to reach forward, bending at the hip with one hand on top of the other, and recording the distance (cm) from the tips of the middle fingers to the top of the toes [22]. If participants could not reach their toes the distance was recorded as a negative number, and if they could reach past their toes, the distance was recorded as a positive number. Waist circumference was measured using a tape measure at the narrowest point of the torso, or at the midpoint between lowest rib and top of iliac crest if the narrowest point was not apparent [23]. All five tests have demonstrated moderate to excellent inter- and intra-rater reliability and criterion validity, in community-dwelling adults [24], and older adults [20, 25–28].

Pre and post-test data were collected by the same individuals as those who delivered the exercise classes. Scores were uploaded to the ERAA portal immediately after testing and then locked for editing. When performing post-test assessments, providers and participants were not blinded to pre-test scores. Additional demographic details collected by providers at the initial assessment included date of birth, gender, postcode of residence and self-reported comorbidities (yes/no) including arthritis, asthma, cancer, dementia, depression, diabetes, heart disease, hypertension, osteoporosis, and prostate issues (males only) (Additional file 1). Postcode of residence was mapped to the Accessibility/Remoteness Index of Australia (ARIA) (a geographical index of remoteness) and the Index of Relative Socio-economic Advantage and Disadvantage (IRSAD, an index of economic and social conditions of people and households within an area) [29]. Comorbidity was categorised as < 2 or ≥ 2 reported comorbidities for analysis.

### Statistical analysis

The sample size calculation was based on the two primary outcomes: STS and TUG. For the STS, minimal clinically important difference (MCID) values are reported to range from 2.0 to 2.6 sit to stand repetitions, with standard deviations ranging from 3.2 to 4.0 [20, 30, 31]. Taking the more conservative values, a minimum sample size of 100 participants were required to detect a pre-post-test difference of 2.0 STS repetitions (SD = 4.0) with a two-sided significance level of 1% and power of 99%. For the TUG, MCID values reported in the literature range from 2.9 to 4.9 s, with standard deviations ranging from 2.0 to 3.0 [25, 32, 33]. Taking the more conservative values, a minimum sample size of 30 participants were required to detect a pre-post-test difference of 2.9 s (SD = 3.0) with a two-sided significance level of 1% and power of 99%. Taking the larger sample size of 100 participants (needed for the STS), and to allow for 50% drop out, a minimum of 200 participants were required for recruitment.

Only those participants with at least partial follow up (i.e. pre and post data available for at least one primary outcome) were included in the primary analysis. Characteristics of these participants were summarised using counts and percentages for categorical variables, and medians and interquartile ranges (for continuous characteristics with skewed outcomes), and compared with the characteristics of participants lost to follow-up using Chi-square or Wilcoxon rank sum analyses.

Primary and secondary outcomes assessed at pre- and post-intervention were reported as mean, SD and range and subsequently modelled to evaluate the change over time using linear mixed-effects regression with patient as a random effect. For primary outcomes, differences in the magnitude of change in score for six key covariates (age group, gender, comorbidity, ARIA classification, IRSAD quintile and class attendance (n/12)) were examined using six separate models that included an interaction term between each variable and time (i.e. pre-test/post-test). Model estimates were reported for any variables with a significant interaction with time (p < 0.05) with the mean change representing the improvement in outcome for each category relative to the reference category. Models were compared with and without clustering by state. Given no significant difference in models and minimal clustering within states (residual intraclass correlation without clustering = 0.76; with clustering = 0.75), the simpler models without clustering were reported. Residual plots were inspected to evaluate model assumptions (i.e. normal distribution of residuals and equal variances). All analyses were performed using Stata v.17, with p < 0.05 considered statistically significant.

## Results

**Fig. 1:**
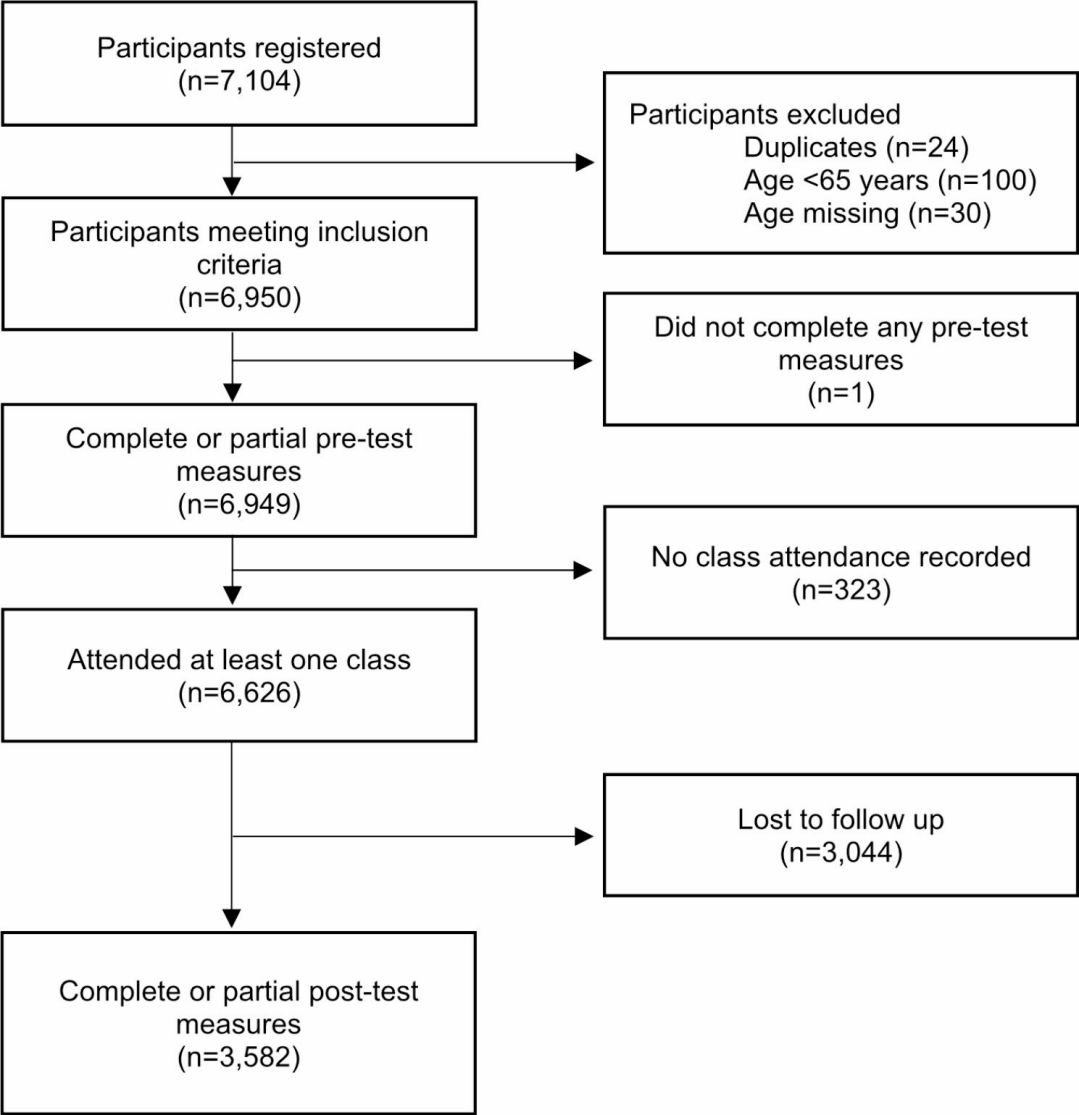
Flow chart of participant inclusion and follow-up

A total of 7,104 participants were initially screened for participation in the Exercise Right for Active Ageing program and 6,950 met the inclusion criteria. Of these individuals, 6,626 attended at least one class (95%) and 3,582 completed or partially completed follow-up testing (52%) (Fig. 1). Of included participants (n = 3,582), the majority were women (77%), were aged between 65 and 74 years of age (63.7%), resided in major cities (62.1%) and reported two or more comorbidities (59.4%) (Table 1). The state with the highest proportion of participants was Queensland (31.4%) and the highest proportion of participants attended a ‘general class’ (61.2%). A total of 3,044 participants (45.9%) were lost to follow-up and they differed significantly to included participants (Table 1). Notably, participants lost to follow-up were slightly older, came from the ACT, NSW, Queensland or Victoria, were residing in inner/outer regional or remote areas, had greater socio-economic disadvantage, had more comorbidities and attended fewer exercise classes. There was also a higher loss to follow up for participants attending classes categorised as aerobics, falls prevention/balance, strength, and ‘other’, than for remaining class types.

**Table 1:** Characteristics of participants included^1^ (n = 3,582) and lost to follow-up (n = 3,044)

Characteristic		Included n (%)	Lost to follow-up n (%)	p
Age (years)	Median (IQR)	72 (69–77)	73 (69–77)	0.003
Age group	65–69 years	1,118 (31.2)	901 (29.6)	0.005
	70–74 years	1,165 (32.5)	920 (30.2)	
	75–79 years	757 (21.1)	700 (23.0)	
	80–84 years	387 (10.8)	344 (11.3)	
	85 + years	155 (4.3)	179 (5.9)	
Gender	Men	822 (23.0)	690 (22.7)	0.874
	Women	2,758 (77.0)	2,353 (77.3)	
	Non-binary	< 5	< 5	
State or Territory^2^	Australian Capital Territory	49 (1.4)	75 (2.5)	< 0.001
	New South Wales	980 (27.4)	1,019 (33.6)	
	Northern Territory	0 (0.0)	< 5	
	Queensland	1,124 (31.4)	1,028 (33.9)	
	South Australia	198 (5.5)	150 (4.9)	
	Tasmania	130 (3.6)	78 (2.6)	
	Victoria	289 (8.1)	304 (10.0)	
	Western Australia	811 (22.7)	376 (12.4)	
ARIA^3^	Major cities	2,220 (62.1)	1,694 (55.7)	< 0.001
	Inner Regional	1,090 (30.5)	1,059 (34.9)	
	Outer Regional	223 (6.2)	234 (7.7)	
	Remote/very remote	44 (1.2)	52 (1.7)	
IRSAD quintile^4^	1 (Most disadvantaged)	284 (7.8)	284 (9.4)	< 0.001
	2	496 (13.9)	615 (20.3)	
	3	883 (24.7)	820 (27.0)	
	4	902 (25.2)	657 (21.6)	
	5 (Most advantaged)	1008 (28.2)	661 (21.8)	
Comorbidities	Low (< 2)	1,456 (40.7)	1,105 (36.3)	< 0.001
	High (2+)	2,126 (59.4)	1,939 (63.7)	
Classes attended	Median (IQR)	12 (12–12)	7 (3–10)	< 0.001
Class type^5^	Aerobics^6^	137 (3.8)	151 (5.0)	< 0.001
	Falls prevention/balance	378 (10.6)	387 (12.7)	
	Low intensity^7^	161 (4.5)	105 (3.1)	
	Strength	225 (6.3)	238 (7.8)	
	Clinical program/condition- specific	389 (10.9)	212 (7.0)	
	General^8^	2,213 (61.8)	1,845 (60.6)	
	Other^9^	78 (2.2)	106 (3.5)	
Total		3,582 (54.1)	3,044 (45.9)	

ARIA, Accessibility/Remoteness Index of Australia; IRSAD, Index of Relative Socio-economic Advantage and Disadvantage

^1^ Defined as pre and post data collected for at least one outcome measure; ^2^ Missing data, n = 11; ^3^ Missing data, n = 10; ^4^ Missing data, n = 16; ^5^ Missing data, n = 1; ^6^ Aerobics includes aerobics, cardiovascular, aqua aerobics/hydro; ^7^ Low intensity includes Pilates equipment/matwork, Yoga, flexibility, mobility; ^8^ General includes circuit class, functional fitness, general fitness, group class, gym-based program; ^9^ ‘Other’ includes walking groups, Tai-Chi, chair-based, small equipment, bush walking, low-impact classes

For all primary and secondary outcomes, there was a statistically significant improvement from pre-test to post-test (p < 0.001) (Table 2). Covariates associated with the two primary outcomes (STS and TUG) were assessed further using linear mixed models. For the STS test, the magnitude of improvement from pre-test to post-test differed by age, gender and IRSAD quintile (i.e. these were the only significant predictors) (Table 3). While there was a significant improvement across all age groups, compared to the reference group (65–69 years), the magnitude of improvement in sit-to-stands was significantly less for participants aged 70 + years (0.3 to 0.8 fewer sit-to-stands). For gender, there was a significant improvement for males and females, but compared to the reference group (males), the magnitude of improvement in sit-to-stands was significantly greater for females (0.4 more sit-to-stands). There was a significant improvement for all quintiles of the IRSAD, but compared to the reference group (most disadvantaged) there was less improvement in the most advantaged group (0.5 fewer sit-to-stands). For the TUG, the magnitude of improvement from pre-test to post-test differed by age group, comorbidity, ARIA classification and IRSAD quintile (Table 4). While there was a significant improvement across all age groups, compared to the reference group (65–69 years), the magnitude of improvement in sit-to-stands was significantly greater for participants aged 80–84 years (0.3 s faster). There was a significant improvement in both comorbidity groups but, compared to the reference group (reporting < 2 comorbidities), the magnitude of improvement for the TUG was significantly greater for people reporting 2 + comorbidities (0.3 s faster). For ARIA classification, there was a significant improvement for all groups but, compared to the reference group (major cities), the magnitude of improvement for the TUG was significantly greater for participants residing in outer regional areas (0.5 s faster). There was a significant improvement for all quintiles of the IRSAD, but compared to the reference group (most disadvantaged) there was less improvement in the most advantaged group (0.4 s slower).

**Table 2:** Pre and post-test values and unadjusted mean differences (linear mixed-effects regression)

	Pre-test	Post-test	β (95% CI) for unadjusted mean difference	P
**Sit to stand (n)** ^1^				
Mean (SD)	11.6 (3.9)	13.7 (4.4)	2.2 (2.1, 2.3)	< 0.001
Range	0.0–34.0	0.0–37.0		
**3 m timed up and go (s)** ^2^				
Mean (SD)	8.3 (3.4)	7.4 (3.0)	-0.9 (-1.0, -0.8)	< 0.001
Range	3.0–47.0	3.0–58.0		
**Grip strength (L) (kg)** ^3^				
Mean (SD)	21.9 (8.2)	23.4 (8.1)	1.5 (1.3, 1.6)	< 0.001
Range	0.0–74.2	0.0–76.0		
**Grip strength (R) (kg)** ^4^				
Mean (SD)	23.5 (8.4)	24.8 (8.4)	1.3 (1.2, 1.5)	< 0.001
Range	0.0–83.6	0.0–73.0		
**Sit and reach (L) (cm)** ^5^				
Mean (SD)	-1.3 (12.6)	0.4 (11.9)	1.7 (1.4, 2.0)	< 0.001
Range	-47.5–49.0	-48.0–50.0		
**Sit and reach (R) (cm)** ^6^				
Mean (SD)	-1.1 (12.7)	0.5 (12.0)	1.7 (1.4, 2.0)	< 0.001
Range	-43.0–50.0	-48.5–50.0		
**Waist circumference (cm)** ^7^				
Mean (SD)	98.3 (14.4)	97.0 (14.2)	-1.2 (-1.4, -1.1)	< 0.001
Range	54.0–173.0	60.0–160.0		

^1^ n = 3497; ^2^n=3374 ^3^n=3381; ^4^n=3400; ^5^ n = 3394; ^6^n=3372; ^7^n=3468

**Table 3:** Predictors of sit to stands and the change in sit to stands from pre-test to post-test (linear mixed-effects regression models)

	Mean (SD) sit to stands		β (95% CI) for adjusted mean difference in sit to stands, from pre-test to post-test	p	β (95% CI) for adjusted mean difference in sit to stands, relative to reference group	p ^1^
	Pre-test	Post-test				
**Age group (years)**						< 0.001
65–69	12.3 (3.9)	14.8 (4.5)	2.5 (2.3, 2.7)	< 0.001	Ref.	
70–74	11.8 (3.6)	14.0 (4.2)	2.2 (2.0, 2.4)	< 0.001	-0.3 (-0.5, -0.1)	0.02
75–79	11.2 (3.8)	13.2 (4.4)	1.9 (1.7, 2.2)	< 0.001	-0.6 (-0.9, -0.3)	< 0.001
80–84	10.6 (3.7)	12.3 (4.0)	1.7 (1.4, 2.0)	< 0.001	-0.8 (-1.1, -0.5)	< 0.001
85+	9.2 (3.5)	11.0 (3.7)	1.8 (1.4, 2.3)	< 0.001	-0.7 (-1.2, -0.2)	< 0.01
**Gender**						< 0.01
Male	11.9 (4.3)	13.8 (4.9)	1.9 (1.7, 2.1)	< 0.001	Ref.	
Female	11.5 (3.7)	13.7 (4.2)	2.3 (2.2, 2.4)	< 0.001	0.4 (0.2, 0.6)	< 0.01
Non-binary	14.5 (0.7)	16.0 (1.4)	1.5 (-2.5, 5.5)	0.47	-0.4 (-4.4, 3.7)	0.86
**IRSAD quintile**						< 0.001
1 (Most disadvantaged)	11.0 (3.7)	13.4 (4.4)	2.4 (2.0, 2.7)	< 0.001	Ref.	
2	11.3 (3.7)	13.4 (4.1)	2.2 (2.0, 2.5)	< 0.001	-0.1 (-0.6, 0.3)	0.53
3	11.6 (3.8)	13.9 (4.2)	2.3 (2.1, 2.5)	< 0.001	0.0 (-0.4, 0.4)	0.93
4	12.1 (3.8)	14.4 (4.1)	2.3 (2.1, 2.5)	< 0.001	0.0 (-0.4, 0.4)	0.95
5 (Most advantaged)	11.4 (4.1)	13.3 (4.6)	1.8 (1.7, 2.0)	< 0.001	-0.5 (-0.9, -0.1)	0.01

CI, confidence interval; IRSAD, Index of Relative Socio-economic Advantage and Disadvantage

^1^ p value for the interaction of this variable with time (i.e. pre-test/post-test)

**Table 4:** Predictors of Timed Up and Go (s) and the change in Timed Up and Go (s) from pre-test to post-test (linear mixed-effects regression models)

	Mean (SD) Timed Up and Go (s)		β (95% CI) for adjusted mean change in TUG (s), from pre-test to post-test	p	β (95% CI) for adjusted mean difference in TUG (s), relative to reference group	p ^1^
	Pre-test	Post-test				
**Age group (years)**						0.01
65–69	7.4 (2.4)	6.5 (1.9)	-0.9 (-1.0, -0.7)	< 0.001	Ref.	
70–74	7.8 (2.3)	7.0 (2.5)	-0.8 (-0.9, 0.7)	< 0.001	0.1 (-0.1, 0.3)	0.36
75–79	8.8 (4.0)	7.8 (3.3)	-1.0 (-1.2, -0.9)	< 0.001	-0.2 (-0.4, 0.1)	0.13
80–84	10.0 (4.1)	8.8 (2.9)	-1.2 (-1.4, -1.0)	< 0.001	-0.3 (-0.6, -0.1)	0.02
85+	11.9 (5.7)	10.8 (5.8)	-1.1 (-1.4, -0.8)	< 0.001	-0.2 (-0.6, 0.1)	0.21
**Comorbidities**						< 0.001
Low (< 2)	7.7 (3.1)	6.9 (2.6)	-0.7 (-0.9, -0.6)	< 0.001	Ref.	
High (2+)	8.7 (3.5)	7.7 (3.2)	-1.0 (-1.1, -0.9)	< 0.001	-0.3 (-0.4, -0.1)	< 0.001
**ARIA**						< 0.01
Major cities	8.1 (3.1)	7.3 (2.9)	-0.8 (-0.9, -0.7)	< 0.001	Ref.	
Inner Regional	8.4 (3.4)	7.5 (3.0)	-1.0 (-1.1, -0.8)	< 0.001	-0.1 (-0.3, 0.0)	0.08
Outer Regional	8.9 (5.0)	7.6 (3.4)	-1.3 (-1.6, -1.1)	< 0.001	-0.5 (-0.8, -0.2)	< 0.01
Remote/Very remote	8.5 (2.6)	7.2 (2.1)	-1.3 (-1.9, -0.7)	< 0.01	-0.5 (-1.1, 0 0.1)	0.13
**IRSAD quintile**						< 0.01
1 (Most disadvantaged)	9.3 (4.1)	8.2 (3.4)	-1.1 (-1.3, -0.8)	< 0.001	Ref.	
2	8.5 (3.1)	7.4 (2.6)	-1.0 (-1.2, -0.9)	< 0.001	0.0 (-0.3, 0.3)	0.95
3	8.4 (3.5)	7.4 (3.1)	-1.0 (-1.2, -0.9)	< 0.001	0.0 (-0.3, 0.3)	0.85
4	8.1 (3.3)	7.2 (3.0)	-0.9 (-1.1, -0.8)	< 0.001	0.1 (-0.1, 0.4)	0.34
5 (Most advantaged)	8.0 (3.1)	7.3 (2.9)	-0.7 (-0.8, -0.6)	< 0.001	0.4 (0.1, 0.6)	0.01

CI, confidence interval; ARIA, Accessibility/Remoteness Index of Australia TUG, Timed Up and Go; IRSAD, Index of Relative Socio-economic Advantage and Disadvantage

^1^ p value for the interaction of the variable with time (i.e. pre-test/post-test)

## Discussion

The aims of this quasi-experimental study were to determine whether Australians aged 65 years and older, who participated in the nationwide Exercise Right for Active Ageing program experienced a change in physical function, and to identify factors associated with this change. For participants who completed follow-up testing, there was a statistically significant improvement in physical function, as indicated by the 30 s Sit-to-Stand test (STS) and the 3-metre Timed Up and Go test (TUG). There were also significant improvements in hand grip strength, lower body flexibility, and waist circumference. For the STS test, there were greater improvements observed in younger age groups, women and those with greater socio-economic disadvantage, while for the TUG test, there were greater improvements in those reporting more comorbidities, and living in outer regional areas and areas with greater socio-economic disadvantage.

Although there were statistically significant improvements across all outcomes, the clinical significance of these improvements must be considered, particularly in the context of such a large sample. For the STS test, which is an indicator of lower limb strength and ADL performance [20], the average magnitude of improvement of 2.2 sit to stand repetitions was within the range of clinically important values (MCID values range from 2.0 to 2.6) [30, 31]. However, for the TUG test, an indicator of functional mobility and dynamic balance [21], the reported improvement of 0.9 s was considerably less than that which is considered clinically important (2.9 to 4.9 s) [32, 33]. It is possible that, in the short-term, strength and ADLs are more responsive to training, than mobility and balance. Furthermore, there is evidence that greater improvements in balance are achieved with a higher dosage of exercise, of up to three hours per week, via classes that specifically aim to challenge balance [6]. While beyond the scope of this study, it would be worthwhile to investigate whether the subgroup of ~ 400 participants who attended falls prevention and balance classes did achieve clinically important improvements in this domain.

In relation to the secondary outcomes of grip strength, lower body flexibility, and waist circumference, improvements were once again small, but statistically significant. For hand grip strength, there is no clear MCID reported in the literature, with changes of 5.0 to 6.5 kg providing an estimate of meaningful change [34]. The reported improvements in grip strength of 1.3 to 1.5 kg fall well short of this range. To our knowledge, there is no reported MCID for the chair sit and reach test, an indicator of lower body flexibility. However, normative values for adults aged 60 + years are − 1.75 cm for men and + 3.25 cm for women [35]. Mean post-test scores in our cohort (+ 0.6 to + 0.7 cm) were better than normative values for men but worse than normative values for women. Similarly, for waist circumference there is no clear MCID reported in the literature. However, the mean reduction of 1.2 cm was less than the 4 cm cited as potentially clinically relevant for people who are overweight or obese [36]. It is also important to note that minimal detectable change values of 1.7 cm for the chair sit and reach test and 3 cm for waist circumference have been reported [37, 38]. As such, mean differences for these outcomes should be interpreted with caution as they lie close to, or within, the magnitude of potential measurement error.

The study also sought to understand factors associated with changes in physical function. Similar to previous research, the magnitude of improvement in the STS test was less for people aged over 70 years, compared to people aged 65–69 years [39]. This outcome was unsurprising given that the physiological effects of ageing are associated with diminishing strength and mobility gains in response to exercise [4]. As such, people in the oldest age groups often need a greater intensity, frequency and duration of exercise in order to make comparable improvements. Women made greater gains than men in the STS test, which was largely accounted for by lower baseline scores. Previous research has reported mixed results on the impact of exercise on physical function according to gender [40]. However, one explanation for the gender differences found in this study is that, because men commonly have greater muscle mass than women, they need a higher amount and/or intensity of exercise to achieve the same benefits [40]. People living in areas with greater socio-economic advantage had less improvement in the STS test than those in the most disadvantaged areas, potentially due to their higher baseline scores. For the TUG test, greater improvements were made by people reporting more comorbidities and those living in outer regional and disadvantaged areas. It is possible that these participants benefitted more from the program than those with fewer comorbidities, and those living in major cities and areas with greater socio-economic advantage. However, it is also important to note that there was a higher loss to follow-up in the high comorbidity, regional and socially disadvantaged groups, which may indicate potential selection bias, with those who were retained being more motivated to participate in the program, resulting in better performance. It is notable that improvements in physical function were not associated with the number of classes attended. However, as reported previously, adherence to the program was high, with a median class attendance of 100% [41]. It was also evident that state of residence did not impact upon physical performance outcomes. This was despite the fact that class participation was lower in states most impacted by COVID-19 lockdowns throughout 2020 and 2021 [41].

There are certain study limitations to acknowledge. Most importantly, being a pre-post study with no control group, this study cannot definitively attribute improvements in physical function to program participation. Also, almost half of the sample were lost to follow up and there were significant differences between participants lost to follow up and those included in the study. Similar to many other studies conducted throughout 2020 and 2021, COVID-19 was likely to have impacted upon program completion and follow-up, particularly in the states of Victoria and NSW where lockdown conditions were imposed for extended periods [42]. Nonetheless, the substantial loss to follow-up is a threat to the external validity of findings. In addition, because assessors and participants were not blinded to participants’ pre-test scores at follow-up, participants may have strived to better their pre-test scores, thereby inflating the effect of the intervention. Finally, given evidence of positive effects of all types of physical activity and exercise programs on physical function in older adults [7], it was not the intention of the study to analyse the impact of specific types of classes. However, it would be beneficial in future research to investigate the impact of different types of exercise classes included in this study.

## Conclusions

In conclusion, participation of older Australians in the Exercise Right for Active Ageing program, led to statistically significant improvements in all physical function outcomes, and clinically important improvements in the STS test, an indicator of lower limb strength and ADL performance. While there was substantial loss to follow-up throughout the study, particularly in states impacted by COVID-19 lockdowns [43], the program reached a large number of older Australians from every state and territory, which supports the feasibility and acceptability of AES- and AEP-led exercise classes amongst community dwelling older adults. Importantly, classes were attended by people traditionally reporting poorer levels of physical function, including those aged over 85 years, with high levels of comorbidity [44]. Classes were also well attended by large numbers of older adults from regional and remote parts of Australia, who report difficulties accessing suitable exercise programs and traditionally have lower levels of exercise engagement [45, 46].

### Electronic supplementary material

Below is the link to the electronic supplementary material.

Supplementary Material 1

## Data Availability

The datasets used and/or analysed during the current study are available from the corresponding author on reasonable request.

## Abbreviations

ACT: Australian Capital Territory
ADLs: Activities of daily living
AEP: Accredited exercise physiologists
AES: Accredited exercise scientists
ANZCTR: Australian New Zealand Clinical Trials Registry
APSS: Adult Pre-Exercise Screening System
ARIA: Accessibility/Remoteness Index of Australia
ERAA: Exercise Right for Active Ageing program
ESSA: Exercise & Sports Science Australia
IRR: Incidence Rate Ratio
MCID: Minimal clinically important difference
MET: Metabolic equivalent of task
NSW: New South Wales
NT: Northern Territory
SA: South Australia
STS: 30 s Sit-to-Stand test
TREND: Transparent Reporting of Evaluations with Nonrandomized Designs guidelines
TUG: 3-metre Timed Up and Go test
WA: Western Australia
WHO: World Health Organization
