# Supplementary material for: Impact of the Exercise Right for Active Ageing program on physical function in older adults: a quasi-experimental pre-post study
Source: BMC Geriatr. 2023 Dec 4;23:799. doi: 10.1186/s12877-023-04499-5 (PMC10696865; doi:10.1186/s12877-023-04499-5)
Supplement: Supplementary file 1 — Supplementary Material 1 [file 12877_2023_4499_MOESM1_ESM.pdf]

Additional file 1. Screening and assessment form

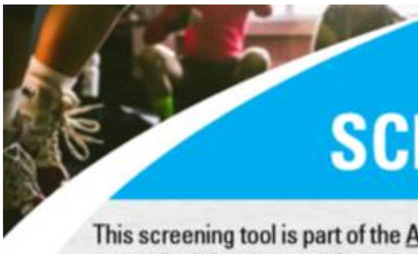

ADULT PRE-EXERCISE SCREENING SYSTEM (APSS)

This screening tool is part of the Adult Pre-Exercise Screening System (APSS) that also includes guidelines (see User Guide) on how to use the information collected and to address the aims of each stage. No warranty of safety should result from its use. The screening system in no way guarantees against injury or death. No responsibility or liability whatsoever can be accepted by Exercise & Sport Science Australia, Fitness Australia, Sports Medicine Australia or Exercise is Medicine for any loss, damage, or injury that may arise from any person acting on any statement or information contained in this system.

Full Name: ______________________

Date of Birth: ______________ Male: ☐ Female: ☐ Other: ☐

STAGE 1 (COMPULSORY)

AIM: To identify individuals with known disease, and/or signs or symptoms of disease, who may be at a higher risk of an adverse event due to exercise. An adverse event refers to an unexpected event that occurs as a consequence of an exercise session, resulting in ill health, physical harm or death to an individual.

This stage may be self-administered and self-evaluated by the client. Please complete the questions below and refer to the figures on page 2. Should you have any questions about the screening form please contact your exercise professional for clarification.

Please tick your response	YES	NO
1. Has your medical practitioner ever told you that you have a heart condition or have you ever suffered a stroke?		
2. Do you ever experience unexplained pains or discomfort in your chest at rest or during physical activity/exercise?		
3. Do you ever feel faint, dizzy or lose balance during physical activity/exercise?		
4. Have you had an asthma attack requiring immediate medical attention at any time over the last 12 months?		
5. If you have diabetes (type 1 or 2) have you had trouble controlling your blood sugar (glucose) in the last 3 months?		
6. Do you have any other conditions that may require special consideration for you to exercise?		

IF YOU ANSWERED 'YES' to any of the 6 questions, please seek guidance from an appropriate allied health professional or medical practitioner prior to undertaking exercise.

IF YOU ANSWERED 'NO' to all of the 6 questions, please proceed to question 7 and calculate your typical weighted physical activity/exercise per week.

7. Describe your current physical activity/exercise levels in a typical week by stating the frequency and duration at the different intensities. For intensity guidelines consult figure 2.

**Intensity**	**Light**	**Moderate**	**Vigorous/High**
**Frequency** (number of sessions per week)			
**Duration** (total minutes per week)			

Weighted physical activity/exercise per week

Total minutes = (minutes of light + moderate) + (2 x minutes of vigorous/high)

TOTAL = ________ **minutes per week**

- If your total is less than 150 minutes per week then light to moderate intensity exercise is recommended. Increase your volume and intensity slowly.
- If your total is more than or equal to 150 minutes per week then continue with your current physical activity/exercise intensity levels.

- It is advised that you discuss any progression (volume, intensity, duration, modality) with an exercise professional to optimise your results.

I believe that to the best of my knowledge, all of the information I have supplied within this screening tool is correct.

Client signature: ______________________ Date: ______________

Participant data entry

First name:		Last name:	
DOB:		Gender:	Female Male Intersex
Postcode:		Email:	
Referral:		Had a free class:	Yes No

Screening done:	Yes No	Screening date:	
Arthritis:	Yes No	Diabetes:	Yes No
Asthma:	Yes No	Heart disease:	Yes No
Cancer:	Yes No	Hypertension:	Yes No
Dementia:	Yes No	Osteoporosis:	Yes No
Depression:	Yes No	Prostate (males):	Yes No

		Consent signed?	Yes No
Class start date:		Class end date:	

	PRE-TEST RESULTS		POST-TEST RESULTS
Pre-test date:		Post-test date:	
30s sit to stand:	times	30s sit to stand:	times
3m up and go:	sec	3m up and go:	sec
Grip str left:	kg	Grip str left:	kg
Grip str right:	kg	Grip str right:	kg
Sit & reach left:	cm	Sit & reach left:	cm
Sit & reach right:	cm	Sit & reach right:	cm
Waist:	cm	Waist:	cm

Physical activity survey

In the past week, what do you estimate was the *total time in minutes* that you spent:

Walking continuously for 10 minutes or more for fun, exercise or to get from place to place?	minutes
Doing other moderate physical activities for fun, fitness or sport (do not count the walking you listed above)	minutes
Doing vigorous gardening, heavy household work or weight lifting that made you breathe harder or puff?	minutes
**On a total of how many days in the past week did you do any of the activities listed above?**	days
**On average, how many hours a day do you spend sitting? Watch TV, at the computer, driving, reading, crafting**	hours

EuroQol 5D 3L

Please indicate with a tick the statement that best describes your state of health today:

**Mobility**	1. I have no problems walking about	☐
	2. I have some problems walking about	☐
	3. I am confined to bed	☐
**Self-care**	1. I have no problems with self-care	☐
	2. I have some problems washing and dressing myself	☐
	3. am unable to wash or dress myself	☐
**Usual Activities** (study, housework, family or leisure activities)	1. I have no problems performing my usual activities	☐
	2. I have some problems performing my usual activities	☐
	3. I am unable to perform my usual activities	☐
**Pain or discomfort**	1. I have no pain or discomfort	☐
	2. I have moderate pain or discomfort	☐
	3. I have extreme pain or discomfort	☐
**Anxiety / depression**	1. I am not anxious or depressed	☐
	2. I am moderately anxious or depressed	☐
	3. I am extremely anxious or depressed	☐
